# Evidence of Vaccine-related Reassortment of Rotavirus, Brazil, 2008–2010

**DOI:** 10.3201/eid1911.121407

**Published:** 2013-11

**Authors:** Tatiana Lundgren Rose, Marcelle Figueira Marques da Silva, Mariela Martinéz Goméz, Hugo Reis Resque, Maria Yury Travassos Ichihara, Eduardo de Mello Volotão, José Paulo Gagliardi Leite

**Affiliations:** Oswaldo Cruz Institute, Rio de Janeiro, Brazil. (T.L. Rose, M.F.M. Silva, M.M. Gomez, H.R. Resque, E. M. Volotão, J.P.G. Leite); Federal University of Bahia, Salvador, Bahia, Brazil (M.Y.T. Ichihara)

**Keywords:** Rotarix, vaccine, G1P[8] strains, phylogenetic analysis, reassortment, rotavirus, RVA, viruses, Brazil

## Abstract

Analysis of 27 rotavirus strains from vaccinated and unvaccinated children revealed reassortment events in 3 strains: a gene derived from a vaccine; a gene acquired from a circulating strain; and reassortment between circulating strains. Data suggest that the widespread use of this monovalent rotavirus vaccine may introduce vaccine genes into circulating human rotaviruses or vice versa.

Group A rotaviruses (RVAs) are a frequent cause of diarrhea in children. The RVA genome consists of 11 dsRNA segments that encode 6 structural (VP1–VP4, VP6, VP7) and 6 non-structural (NSP1– NSP6) proteins (*1*). The basis of a new classification system is phylogenetic analysis of 11 RVA genome segments, although binary classification is still used; genotyping is based on the coding genes for VP7 (G) and VP4 (P) (*2*).

Vaccination is considered effective in reducing the consequences of RVA. Two vaccines, Rotarix (Glaxo SmithKline, Brentford, UK) and RotaTeq (Merck & Co., Whitehouse Station, NJ, USA), are licensed in several countries. Both vaccines demonstrated broad protection against the most common RVA genotypes (*3*).

In Brazil, Rotarix, a monovalent attenuated human rotavirus vaccine for infants 6–24 weeks of age, was introduced in the National Immunization Programs in March, 2006. The vaccine is delivered in 2 doses, >4 weeks apart. In 2009, when vaccine coverage achieved 85.9%, reduction in hospitalization associated with diarrhea (17%) and in related mortality rates (22%) was observed (*4*). This study aimed to assess phylogenetic relationships between G1P[8] RVA obtained from vaccinated and unvaccinated children hospitalized for acute gastroenteritis.

## The Study

During 2008–2010, fecal samples were collected from 3,852 children; 702 specimens (18.2%) had RVA-positive ELISA results; 27 of those (3.8%) were characterized as G1P[8] by using reverse transcription-PCR. Eighteen of the 27 specimens were from vaccinated children (Table 1). Study methods were approved by Fiocruz Ethical Committee (No. 311/06).

**Table 1:** Clinical features of 27 patients infected with group A rotaviruses (G1P[8]), Brazil 2008–2010*

Strain	State	Age (mo)†	Rotarix doses (mo/yr)		Time elapsed between vaccination and hospitalization	Clinical symptoms			
			1st	2nd		Diarrhea‡	Blood	Fever	Vomit
ES15221–08	ES	42	–	–	–	–	–	–	–
SE15901–08	SE	2	09/2008	–	7 d	3	No	No	Yes
SE 16536–09	SE	2	–	–	–	2	No	Yes	Yes
SE 16537–09	SE	4	03/2009	–	2 mo	6	No	Yes	Yes
SE 16779–09	SE	41	03/2006	05/2006	37 mo	1	Yes	Yes	Yes
SE 16782–09	SE	19	02/2008	04/2008	15 mo	3	No	No	y
SE 16800–09	SE	7	02/2009	–	5 mo	3	–	No	No
SE 16803–09	SE	10	11/2008	01/2009	6 mo	1	No	Yes	Yes
SE 16894–09	SE	20	02/2008	05/2008	15 mo	2	No	Yes	No
SE 16897–09	SE	15	08/2008	10/2008	10 mo	1	No	No	Yes
SE 16898–09	SE	4	06/2009	–	2 mo	1	No	No	Yes
SE 16977–09	SE	9	02/2009	04/2009	4 mo	13	No	Yes	Yes
SE 16978–09	SE	10	01/2009	03/2009	6 mo	2	No	Yes	No
SE 17120–09	SE	22	02/2008	05/2008	17 mo	1	No	No	Yes
SE 17122–09	SE	9	07/2008	09/2008	5 mo	2	–	Yes	No
SE 17123–09	SE	8	–	–	–	3	No	Yes	Yes
SE 17241–09	SE	7	06/2008	08/2008	3 mo	2	No	Yes	Yes
PE17887–09	PE	6	–	–	–	–	No	Yes	Yes
PE 17888–09	PE	10	11/2008	–	8 mo	–	No	Yes	Yes
PE 17890–09	PE	20	02/2008	05/2008	18 mo	–	No	No	Yes
PE 17891–09	PE	12	–	–	–	–	No	Yes	Yes
MA18999–10	MA	18	–	–	–	–	–	–	–
MA 19006–10	MA	2	08/10	–	4 d	1	Yes	No	No
MA 19013–10	MA	10	–	–	–	–	–	–	–
MA 19015–10	MA	48	–	–	–	–	–	–	–
MA 19030–10	MA	32	03/2008	–	29 mo	7	Yes	No	No
BA19391–10	BA	2	–	–	–	2	–	No	No

*Prefixes represent origins of circulating strains in Brazil: ES, Espirito Santo; –, no data; SE, Sergipe; PE, Pernambuco; MA, Maranhão; BA, Bahia. †Age of child at time of fecal sample collection. ‡Number of days child had diarrhea before fecal sample collection.

RVA detection, genotyping, and sequencing were performed (*5*). Sequences were deposited in GenBank under accession numbers: JQ926436-JQ926600 and JX683535-JX683664. Sequences were compared with those in strains obtained from GenBank (including Rotarix JX943604.2–JX943614.2).

Strains analyzed belonged to the Wa-like genogroup (genotype 1); 26 strains showed a G1–P[8]–I1–R1–C1–M1–A1–N1–T1–E1–H1 genome constellation. One sample, collected from a child vaccinated with 1 dose during 2010 in Maranhão (MA) state (MA19030–10), contained the G1–P[8]–Ix–R1–Cx–M1–A1–N1–T3–E1–H1 constellation.

Nucleotide (nt) identity values between circulating strains in Brazil and the Rotarix strain ranged 76%–100% (Table 2). Three samples showed 100% nt identity with the Rotarix strain in >1 gene. The SE15901–08 strain, collected on day 7 after the first dose, showed 100% nt identity with all Rotarix strain gene segments and could represent vaccine shedding. Vaccine antigen excretion detected by ELISA achieved 80%, declining to 18%–24% when collected at day 30; 11%–16% of children shed the virus at day 45 (*6*). Shedding is not associated with increased gastroenteritis-like symptoms. Widespread use of Rotarix might reveal adverse reactions not observed in clinical trials, emphasizing the need for global surveillance (*7*).

**Table 2:** Nucleotide similarity values when comparing the Brazilian G1P[8] samples with the Rotarix strain gene segments and genotype constellation of selected human group A rotaviruses*

		Protein encoded by genes†										
Strain names‡	Genotype constellation	VP7	VP4	VP6	VP1	VP2	VP3	NSP1	NSP2	NSP3	NSP4	NSP5
SE15901–09	Vaccine	G1 100%	P[8] 100%	I1 100%	R1 100%	C1 100%	M1 100%	A1 100%	N1 100%	T1 100%	E1 100%	H1 100%
MA19006–10	Vaccine	G1 100%	P[8] 99%	I1 99%	R1 99%	C1 98%	M1 99%	A1 98%	N1 100%	T1 100%	E1 99%	H1 98%
BA19391–10	Vaccine	G1 100%	P[8] 99%	I1 100%	R1 99%	C1 98%	M1 100%	A1 100%	N1 100%	T1 100%	E1 99,6%	H1 100%
ES15221–08	Wa-like (ES)	G1 94%	P[8] 90%	I1 90%	R1 100%	C1 90%	M1 93%	A1 92%	N1 91%	T1 97%	E1 92%	H1 92%
SE16800–09	Wa-like (SE)	G1 94%	P[8] 92%	I1 89%	R1 97%	C1 90%	M1 96%	A1 92%	N1 88%	T1 98%	E1 98%	H1 92%
PE17888–09	Wa-like (PE)	G1 94%	P[8] 92%	I1 90%	R1 94%	C1 91%	M1 93%	A1 93%	N1 90%	T1 97%	E1 94%	H1 93%
MA19013–10	Wa-like (MA)	G1 95%	P[8] 93%	I1 92%	R1 95%	C1 92%	M1 95%	A1 94%	N1 92%	T1 97%	E1 94%	H1 93%
MA19030–10	Wa/AU-1 reassortant	G1 93%	P[8] 91%	X	R1 93%	X	M1 94%	A1 93%	N1 89%	T3 76%	E1 92%	H1 93%
Wa	Wa-like	G1	P[8]	I1	R1	C1	M1	A1	N1	T1	E1	H1
DS-1	DS-1-like	G2	P[4]	I2	R2	C2	M2	A2	N2	T2	E2	H2
AK26	Wa/DS-1 reassortant	G2	P[4]	I2	R2	C2	M2	A2	N1	T2	E2	H2
6809	Wa/DS-1 reassortant	G8	P[6]	I2	R1	C1	M1	A1	N1	T1	E1	H1
Matlab13	Wa/DS-1 reassortant	G12	P[6	I1	R1	C1	M1	A1	T2	T1	E1	H1
Mani-253	Wa/DS-1 reassortant	G4	P[4]	I1	R1	C1	M2	A8	N1	T1	E1	H1
AU-1	AU-1-like	G3	P[9]	I3	R3	C3	M3	A3	N3	T3	E3	H3
T152	AU-1-like	G12	P[9]	I3	R3	C3	M3	A12	N3	T3	E3	H6
K8	Wa/AU-1 reassortant	G1	P[9]	I1	R3	C3	M3	A1	N1	T3	E3	H3
Mani-265	DS-1/AU-1 reassortant	G10	P[6]	I2	R2	C2	M2	A3	N2	T7	E2	H2

*Wa-like, DS-1-like, AU-like genotypes are shown in green, red, and orange, respectively, and the P[6] genotype is shown in blue; X, indeterminate genotypes; –, no data. †One sample for each group was chosen as representative and the average was shown. ‡Prefixes represent origins of circulating strains in Brazil: SE, Sergipe; BA, Bahia; MA, Maranhão; ES, Espirito Santo; PE, Pernambuco.

**Figure 1:**
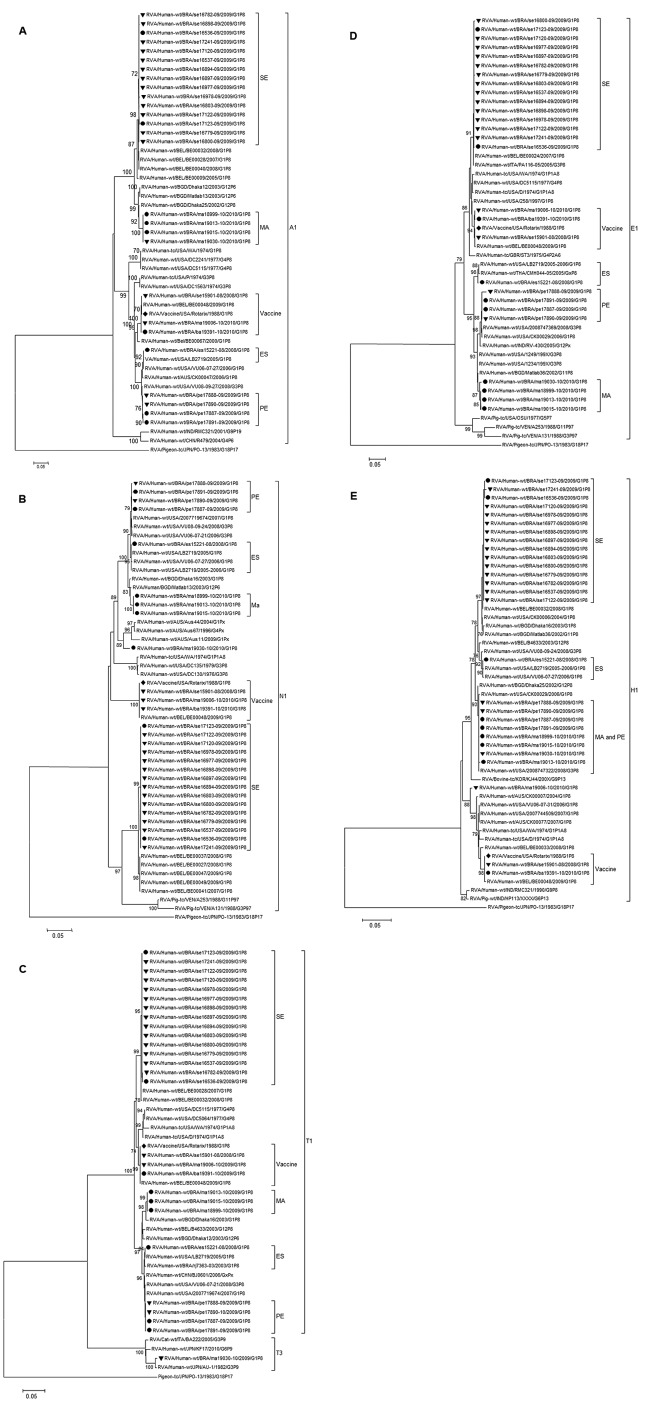
Phylogenetic analysis of nucleotide sequence of A) nonstructural protein 1 [NSP1], B) NSP2, C), NSP3, D) NSP4, and E) NSP5 encoding genes of G1P[8] RVA samples collected in Brazil and described in this study. Filled circles indicate samples from non-vaccinated children and filled triangles vaccinated children. The Rotarix strain is indicated by a filled diamond. Bootstrap values >70%, estimated with 2,000 pseudoreplicate datasets, are indicated at each node. Only the genotype in which the strains under investigation cluster is shown completely; 1 prototype from another genotype has been added as an outgroup. Scale bars represent 0.5 substitutions per nucleotide site.

In phylogenetic analysis of 11 genes, 10 genes clustered into 5 clades. In the remaining gene, NSP5, the circulating strains clustered into 4 groups (Figure 1, panel E).

**Figure 2:**
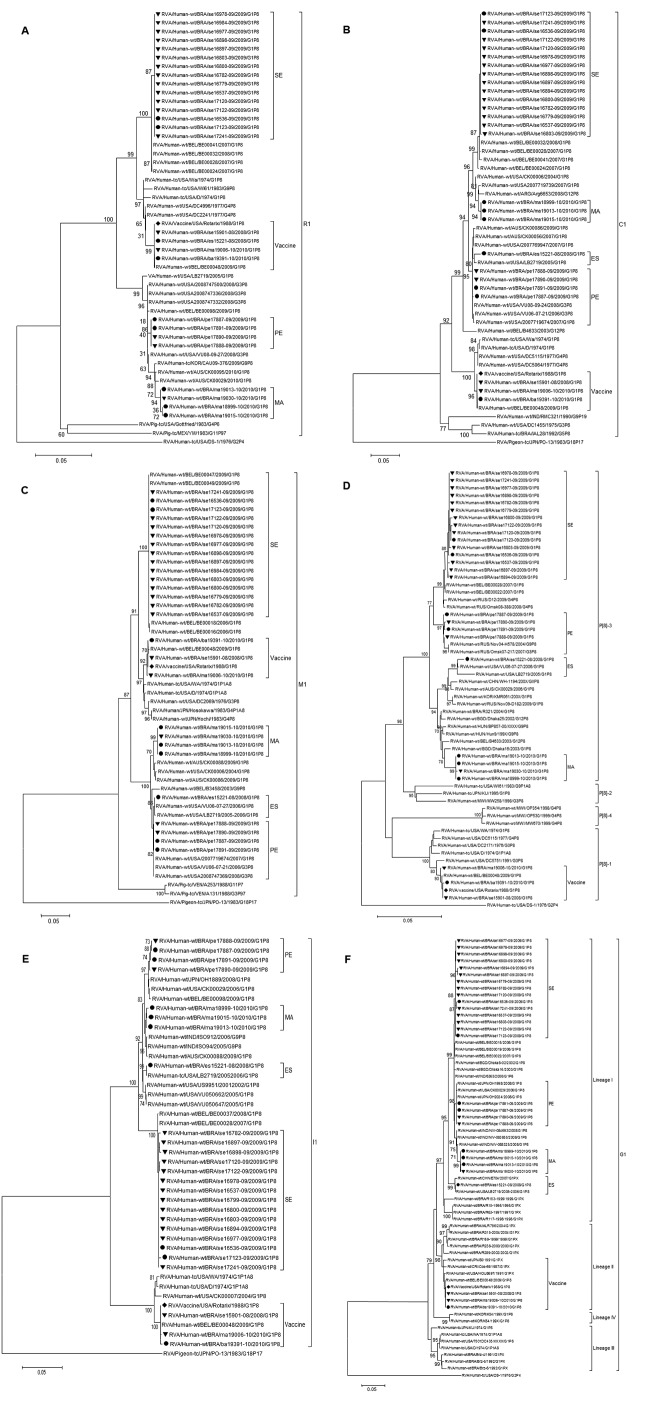
Phylogenetic analysis of nucleotide sequence of (A) VP1, (B) VP2, (C), VP3, (D) VP4, (E) VP6 and (F) VP7 genes of G1P[8] RVA samples collected in Brazil and described in this study. Filled circles indicate samples from non-vaccinated children and filled triangles vaccinated children. The Rotarix vaccine strain is indicated by a filled diamond. Bootstrap values above 70%, estimated with 2,000 pseudoreplicate datasets, are indicated at each node. Only the genotype in which the strains under investigation cluster is shown completely and one prototype from another genotype has been added as outgroup. Scale bars represent 0.5 substitutions per nucleotide site_._

Strain ES15221–08, detected in an unvaccinated child, is genetically distinct and differs in origin from other G1P[8] circulating strains (Figures 1,2). The VP1 gene (648 bp, Figure 2, panel A) in this sample showed 100% nt identity with the Rotarix strain. Strain MA19006–10 was genetically similar to the strain; however, the NSP5 segment (Figure 1, panel E) was closely related to a G1P[8] strain from Australia (JF490152). These 2 strains appear to have been generated by reassortment with this vaccine strain.

Strain MA19030–10, detected in a child after 1 vaccine dose, was closely related to the MA group, but the NSP2 segment differed in origin from other MA samples (Figure 1, panel B) because it clustered with other Wa-like strains. The NSP3 gene belonged to genotype 3 because it was 99.2% (nt) similar to the AU-1 prototype strain (DQ490535.1). These results suggested reassortment events between Wa-like and AU-1 like co–circulating strains.

NSP2 and NSP3 genes from sample MA19030–10 and NSP5 genes from sample MA19006–10 differed from each other and from those of their respective clusters. Samples MA19006–10 and MA19030–10 are genetically distinct and might have distinct evolutionary histories. Studies that included this genogrouping system were performed to prove the existence of inter-genogroup reassortment between human RVA genogroups or human and animal genogroups. The existence and effectiveness of heterogeneous genome constellations remains unclear, probably because it is caused by mechanisms that create protein sets that work better when kept together (*8*).

Phylogenetic analysis of the VP8* (aa 1–247) portion of VP4 encoding gene showed circulating strains (Figure 2, panel D) clustered into 2 lineages (P[8]-3 and P[8]-1). The alignment of the deduced aa sequences showed potential trypsin cleavage sites at arginine 240 and 246 conserved in all samples. All circulating strains contained 91.3%–100% identical aa residues to the Rotarix strain in VP8* antigenic epitopes; no changes were observed on epitopes 8–2 and 8–4.

Two VP7 lineages (G1-I and G1-II) were identified. Samples closely related to the Rotarix strain belonged to G1-II; remaining strains belonged to G1-I. Comparing the regions defined as antigenic epitopes (7–1 and 7–2) for VP7 protein, we found >2 epitopes (aa 94, 123, 148, 217) were not conserved among circulating strains compared with the Rotarix strain. The following amino acids were conserved in the non-VP7/non-VP4 segments of either the Rotarix strain or other strains analyzed: cysteine residues involved in disulfide bonds at positions 6, 8, 85, and 285 in NSP2 protein; the leucine, isoleucine, aspartic acid, methionine and glutamine at positions 275, 281, 288, and 295 in the NSP3 protein; the N-linked glycosylation sites at positions 8 and 18, and cysteine residues at positions 63 and 71 in NSP4 protein; and serine residues at positions 153, 155, 163, and 165 in NSP5 protein.

## Conclusions

This report characterizes the complete genome of G1P[8] strains in Brazil. Phylogenetic analysis showed that sequences clustered consistently with the region of sample collection. Three strains circulating in Brazil were closely related to those in Rotarix; 1 of the 3 was 100% identical to Rotarix, likely representing shedding of this vaccine. Sequence analysis confirmed the presence of the Rotarix VP1–derived segment in 1 sample (ES15221–08), indicating an unreported reassortment event between the vaccine and a community strain.

The backbone of MA19030–10 sample was Wa-like, but the NSP3 segment exhibited a T3 genotype that was described for the AU-1 genogroup. This finding suggests that this strain derived its NSP3 gene from an AU-1-like strain through reassortment.

Changes in antigenic regions of VP4 and VP7 proteins have been associated with mutated RVA strains that spread (*9*,*10*). Comparison of the aa sequences of VP7 and VP8* of strains circulating in Brazil and the monovalent vaccine strain demonstrated that VP7 and VP8* of the circulating strains showed similar antigenic regions to those of the vaccine. No differences between strains from vaccinated and unvaccinated children were observed.

Considering the segmented RVA genome and that the Rotarix vaccine is an attenuated RVA human strain, it is expected that reassortants will arise and circulate among humans. The effects of such events are not known. This study described strains that originated from reassortment events between the Rotarix vaccine strain and strains detected in vaccinated and unvaccinated children. Improvement of RVA surveillance programs that include full genome sequencing analysis will strengthen the understanding of how vaccines will affect the RVAs circulating among humans, and how those events could affect the use of live vaccines, the frequency of RVA intra- and intergenogroup reassortment events under natural conditions, and the stability of RVA generated by such events.
